# The efficacy and safety of anti-CD19/CD20 chimeric antigen receptor- T cells immunotherapy in relapsed or refractory B-cell malignancies:a meta-analysis

**DOI:** 10.1186/s12885-018-4817-4

**Published:** 2018-09-26

**Authors:** Hui Zhou, Yuling Luo, Sha Zhu, Xi Wang, Yunuo Zhao, Xuejin Ou, Tao Zhang, Xuelei Ma

**Affiliations:** State Key Laboratory of Biotherapy and Cancer Center, West China Hospital, Sichuan University and Collaborative Innovation Center, No.37, Guoxue Alley, Chengdu, 610041 People’s Republic of China

**Keywords:** Chimeric antigen receptor T (CAR T) therapy, Safety, Efficacy, Relapsed or refractory B-cell malignancies

## Abstract

**Background:**

Chimeric antigen receptor T (CAR T) cells immunotherapy is rapidly developed in treating cancers, especially relapsed or refractory B-cell malignancies.

**Methods:**

To assess the efficacy and safety of CAR T therapy, we analyzed clinical trials from PUBMED and EMBASE.

**Results:**

Results showed that the pooled response rate, 6-months and 1-year progression-free survival (PFS) rate were 67%, 65.62% and 44.18%, respectively. We observed that received lymphodepletion (72% vs 44%, *P* = 0.0405) and high peak serum IL-2 level (85% vs 31%, P = 0.04) were positively associated with patients’ response to CAR T cells. Similarly, costimulatory domains (CD28 vs CD137) in second generation CAR T was positively associated with PFS (52.69% vs 33.39%, *P* = 0.0489). The pooled risks of all grade adverse effects (AEs) and grade ≥ 3 AEs were 71% and 43%. Most common grade ≥ 3 AEs were fatigue (18%), night sweats (14%), hypotension (12%), injection site reaction (12%), leukopenia (10%), anemia (9%).

**Conclusions:**

In conclusion, CAR T therapy has promising outcomes with tolerable AEs in relapsed or refractory B-cell malignancies. Further modifications of CAR structure and optimal therapy strategy in continued clinical trials are needed to obtain significant improvements.

**Electronic supplementary material:**

The online version of this article (10.1186/s12885-018-4817-4) contains supplementary material, which is available to authorized users.

## Background

**Fig. 1:**
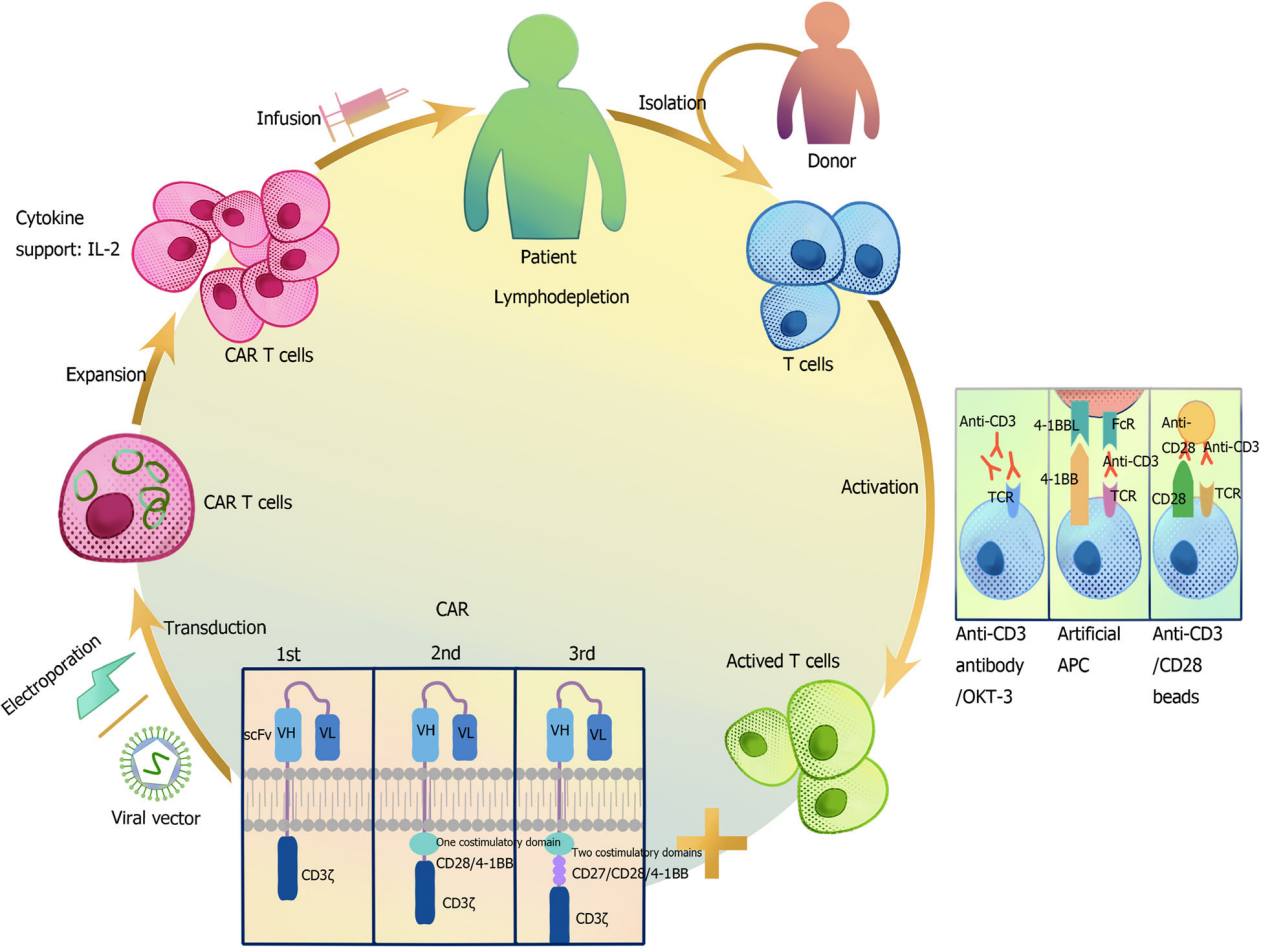
CAR T cell therapy. T lymphocytes from the patient or a suitable donor are isolated. Then T cells are activated with anti-human CD3/CD28 antibody-coated beads, anti-CD3 monoclonal antibodies, and/or artificial antigen-presenting cells(APCs). The first, second or third generation CARs are transducted to T cells via a viral or nonviral vector (i.e., eletroporation). Engineered CAR T cells are expanded and infused into the patient who received or not received lymphodepletion regimen

Recently, chimeric antigen receptor T (CAR T) cells immunotherapy is rapidly developed. Generally, CAR consists of tumor associated antigen (TAA) binding domain, hinge domain, transmembrane domain and signaling domain. TAA usually is a single-chain variable fragment (scFv). Unlike physiological T cell receptors (TCR), scFv can recognize antigen directly without major histocompatibility complex (MHC) restriction. Intracellular signaling domains generally contain immunoreceptor tyrosine-based activation motifs (ITAMs), which usually is CD3ζ and costimulatory molecule (CM), including CD28, CD134 (OX40) and CD137 (4-1BB) [1–4]. T cell activation is initiated through the ITAMs presented in the CD3 polypeptides [5]. The first generation of CAR contains a single signaling domain, usually are CD3ζ chain [6]. Second generation CAR have one signaling domain, and one costimulation domain, with which T cells can expand and functioning under the exist of antigen [1]. Three signaling domains with two costimulatory molecules were engineered to design the third generation CAR. CAR T therapy including the following procedures: first, collecting T cells from the patient or donor; second, isolating and activating T cells [7]; third, modifing T cells with CARs with viral vector transduction or electroporation of RNA or DNA; fourth, expanding the transduced cells; finally, patients receive lymphodepletion and the infusion (Fig. 1).

CD19 is expressed restrictively to B cells, so it is a potential target [8, 9]. CD20 exists in over 90% of B-cell lymphomas and is also used to treat non-Hodgkin’s lymphoma (NHL) [10, 11]. However, there were great difference of efficacy in different trials. Additionally, the efficacy of CAR T cells might be affected by the different execute procedures. However, the critical factors for better efficacy are still unclear.

The adverse effects of CAR T therapy were big challenges, including the cytokine release syndrome(CRS), on-target off-tumor toxicities and toxicities caused by the lymphodepletion chemotherapy [4, 12–14]. Fevers, fatigue and hypotension were often reported [4, 12–14]. However, the most frequently occurred events and the incidence of any treatment adverse events are unknown.

Previous study evaluated the efficacy of anti-CD19 CAR T cells therapy, but it didn’t assess the factors related to progression free survival and the safety of this therapy [15]. The two systematic reviews which estimate efficacy and safety of anti-CD19 CAR T cells therapy were limited because that only 5 and 6 trials were included, respectively [16, 17]. In this study, we aimed to assess the efficacy and safety of CD19 or CD20-CAR T cells immunotherapy. Furthermore, we detected the factors affecting the efficacy and safety of therapy.

## Methods

### Literature searching and inclusion criteria

We searched the PubMed and EMBASE databases for relevant articles published up to September 5, 2016 with the search term “cart”. All studies related to the topics were included. All articles were published in English.

### Literature screening

We extracted the data from each study: first author, year, number of patients, disease type, Ag recognition moieties, costimulatory domains, CART generation, original T cell sources (autologous or allogeneic), T cell culture time, transduction method, T cell treatment, CAR T cells persistence time, lymphodepletion, IL-2 infusion to patients, IL-2 infusion to cells, the infused total cell number, CAR T cells number, peak serum TNF level, peak serum IFN- γ level, peak serum IL-2 level, patients’ response to CAR T therapy, follow-up time and toxicity of the treatments.

There were two outcomes for the efficacy analysis. The primary efficacy outcome was patients’ response rate to CAR T therapy. Patients died not because of the disease or did not evaluate the response were not included for this analysis. The secondary efficacy outcome was patients’ progression free survival (PFS). For the safety analysis, we calculated the occurrence of toxicity of CAR T therapy and observed some frequent adverse events.

### Statistic analysis

We used the Metaprop module in the R-3.3.2 statistical software package to analyze the response rate and the toxicity. Tests of heterogeneity were performed. When the I^2^ statistic was less than 50% and the *p*-value was more than 0.10, results were considered homogenous and a fixed-effect model was used. Otherwise, a random-effect model was used [18]. Subgroup analysis were performed to find the possible predictors.

We used Stata 12.0 to analyze PFS. All the factors analyzed in subgroup analysis of response were evaluated. PFS curves were assessed using the Kaplan–Meier method and compared by the log-rank test in the univariate meta-regression analysis. The independent prognostic factors of PFS was identified by cox regression model.

Contour-enhanced funnel plots was used to assess possible publication bias.

## Results

**Fig. 2:**
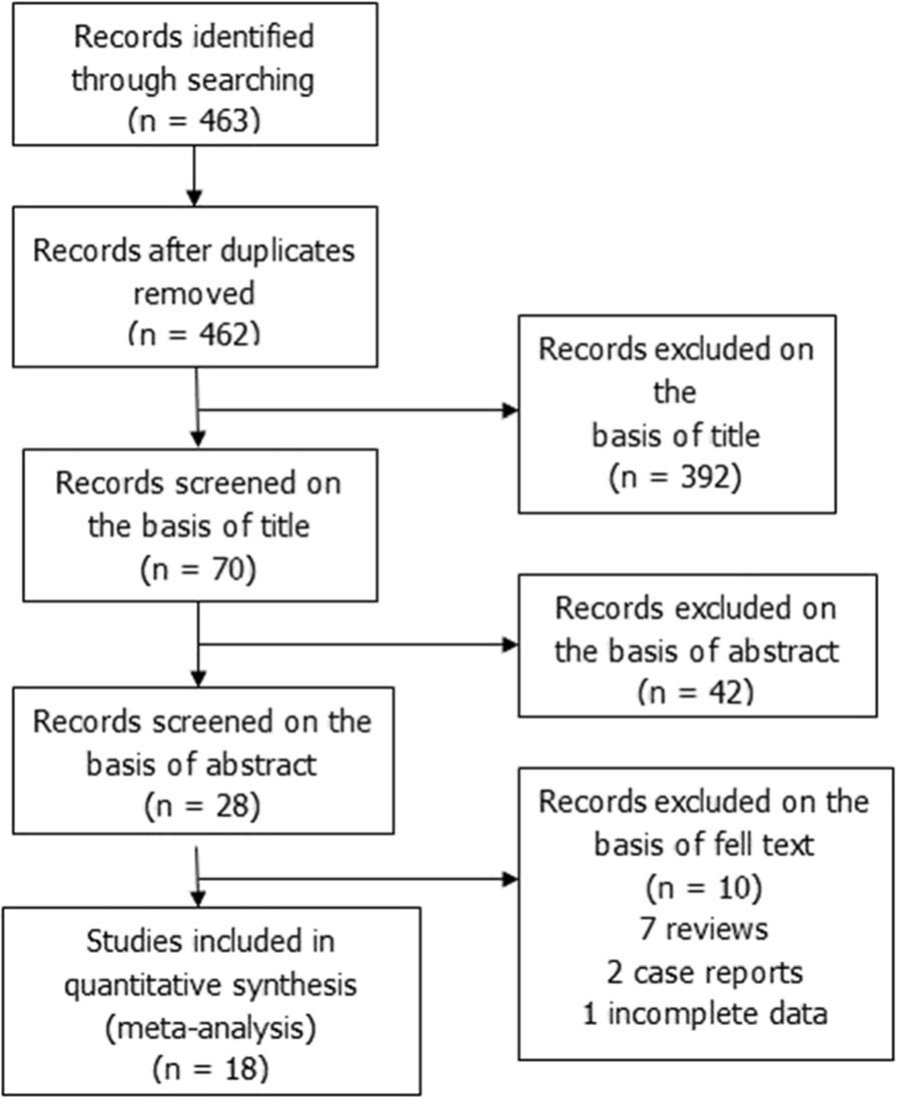
Flow diagram of study selection process

A total of 463 clinical trials were identified by the initial database search. A total of 18 articles were identified for analysis (Fig. 2).

Our study included 18 clinical trials and 185 B cell malignancies patients (126 leukemia and 59 lymphoma) received CAR T cells immunotherapy. The 126 leukemia patients included 39 chronic lymphocytic leukemia patients and 87 acute lymphocytic leukemia patients. The 59 lymphoma patients consisted of 31 diffuse large B-cell lymphoma, 11 mantle cell lymphoma, 7 non-Hodgkon’s lymphoma, 4 follicular lymphoma and 6 patients without detailed subtypes.

### Treatment procedures

The characteristics of CAR T therapy were included in Table 1. Twelve patients in three trials were used with anti-CD20 CAR T. Three patients in one trial received third-generation CAR T with CD28, CD3ζ and CD137 (4-1BB) activation domains. OKT3, rHuIL-2, IL-15, LCL-irradiated, CD3/CD28 beads and CD19/CD80 artificial APCs were added into CAR T cells. The infused CAR T cell number ranged from 1.8 × 10^6^ to 3.2 × 10^9^.

**Table 1:** Clinic trials and patients characteristics

Study	No. of patients	Disease type	Disease status	Ag recognition moieties	costimulatory domains	CART generation	Original T cell sources	T cell culture time	Transduction method	T cell treatment	CAR T cells persistence time	Lymphodepletion	IL-2 infusion to patients	IL-2 infusion to cells	The infused total cell number	CAR T cells number	Patients’ response
Kochenderfer, J. N. (2012) [13]	8	4: lymphoma 4: CLL	Advanced, progressive	CD19	CD28+ CD3ζ	2nd	Autologous	24 days	Gammaretrovirus	OKT3	< 20 days, > 6 months	Cyclophosphamide, fludarabine	Yes	–	0.5–5.5 × 10^7^	0.3–3.0× 10^7^	5 PR 1 CR 1 SD 1 Died with influenza
Jensen, M. C. (2010) [34]	4	2: DLBCL 2: FL	Recurrent, refractory	2: CD20 2: CD19	CD3ζ	1st	Autologous	106 days	Electroporation	OKT3, rHuIL-2	1 day-1 week	BCNU TBI cytoxan, VP-16; Fludarabine	NO; yes	Yes	3–21 × 10^8^; 40–60 × 10^8^/m^2^	–	2 PD 1 Died 1 PR
Kochenderfer, J. N. (2013) [35]	10	4: CLL 4: MCL 2: DLBCL	Progressive	CD19	CD28 CD3ζ	2nd	Allogeneic	8 days	Gammaretrovirus	OKT3, IL-2	1 month	No	No	Yes	1–10 × 10^6^/Kg	0.4–7.8× 10^6^/Kg	6 SD 2 PD 1 CR 1 PR
Brentjens, R. J. (2013) [36]	5	ALL	Relapsed	CD19	CD28 CD3ζ	2nd	Autologous	14 days	Gammaretrovirus	CD3/CD28 beads	3–8 weeks	Cyclophosphamide	No	–	1.2–6.2 × 10^8^	1.4–3.2× 10^8^	5 CR
Till, B. G. (2012) [37]	3	2: MCL 1: FL	Relapsed, refractory	CD20	CD28 CD3ζ CD137 (4-1BB)	3rd	Autologous	> 69 days	Electroporation	OKT3, IL-2	12 months, 9 months	Cyclophosphamide	Yes	–	4.4 × 10^9^/m^2^	–	1 PR 2 NE
Brentjens, R. J. (2011) [11]	9	8: CLL 1: ALL	Relapsed, refractory	CD19	CD28 CD3ζ	2nd	Autologous	11–18 days	Retrovirus	CD3/CD28 beads, CD19/CD80 artificial APCs	0 days, < 4 weeks, > 8 weeks	No; Cyclophosphamide	No	Yes	1.0–11.1 × 10^9^	1.4–32 × 10^8^	3 NR 1 NE 2 PD 2 SD 1 PR
Cruz, C. R. (2013) [38]	8	6: ALL 2: CLL	Relapse	CD19	CD28 CD3ζ	2nd	Allogeneic	40 ± 12 days	Retrovirus	Irradiated LCLs, IL-2	1–12 weeks	–	No	Yes	1.9–11.3 × 10^7^	–	3 CR 1 SD 1 PR 3 PD
Dai, H. (2015) [39]	9	ALL	Relapsed, refractory	CD19	4-1BB CD3ζ	2nd	Autologous; Allogeneic	10–12 days	Lentivirus	OKT3, IL-2	> 6 weeks, < 3–4 weeks	C-MOAD; no	No	Yes	–	2.2–7.9 × 10^8^	3 PD 2 CR 4 PR
Davila, M. L. (2014) [40]	16	ALL	Relapsed, refractory	CD19	CD28 CD3ζ	2nd	Autologous	14 days	Gammaretrovirus	CD3/CD28 beads	2–3 months	Cyclophosphamide	No	–	–	3 × 10^6^/kg	14 PR 2 NR
Savoldo, B. (2011) [28]	6	NHL	Relapsed, refractory	CD19	CD28 CD3ζ; CD3ζ	1st and 2nd	Autologous	6–18 days	Retrovirus	OKT3, IL-2	4–6 weeks; < 6 weeks	–	No	Yes	2–20 × 10^7^/m2	–	4 PD 2 SD
Kochenderfer, J. N. (2015) [32]	15	9: DLBCL 2: lymphoma 4: CLL	Relapsed, refractory	CD19	CD28 CD3ζ	2nd	Autologous	10 days	Gammaretrovirus	OKT3, IL-2	35- > 75 days	Cyclophosphamide, fludarabin	No	Yes	–	1–5 × 10^6^/kg	8 CR 4 PD 1 SD 2 NE
Maude, S. L. (2014) [41]	30	ALL	Relapsed, refractory	CD19	4-1BB CD3ζ	2nd	Autologous	12 days	Lentivirus	CD3/CD28 beads	> 11 months	3: no 15: Flu/Cy 5: Cy/VP 3: Cy 2: CVAD 1: Clofarabine 1: Etoposide/Cytarabine	No	–	–	0.3–9.58 × 10^8^	27 CR 3 NR
Porter, D. L. (2015) [26]	14	CLL	Relapsed, refractory	CD19	4-1BB CD3ζ	2nd	Autologous	10–12 days	Lentivirus	CD3/CD28 beads	1–12 months	3: fludarabine/cyclophosphamide 5: pentostatin/cyclophosphamide 6: bendamustine	No	–	–	0.14–11 × 10^8^	4 CR 4 PR 6 NR
Wang, Y. (2014) [42]	7	DLBCL	Refractory	CD20	CD137 CD3ζ	2nd	Autologous	10–12 days	Lentivirus	–	> 90 days	Cyclophosphamide, Vincristine, Etoposide, Dexamethasone, Doxorubicin, Methylprednisolone, Carboplatin, cytosine, arabinoside; NO	No	–	1–6 × 10^7^/kg	0.2–2.2 × 10^7^/kg	1 CR 1 Died of massive hemorrhage of alimentary tract 4 PR 1 PD
Kochenderfer, J. N. (2010) [43]	1	FL	Progressive	CD19	CD28, CD3ζ	2nd	Autologous	18 days	Retrovirus	IL-2, OKT3	27 weeks	Cyclophosphamide, fludarabin	Yes	Yes	–	4× 10^8^	PR
Wang, X. (2016) [44]	16	7: DLBCL 1: MCL; 4: DLBCL 4: MCL	Relapse	CD19	CD3ζ; CD28 CD3ζ	1st; 2nd	Autologous	7–19 days	Lentivirus	CD3/CD28 beads, IL-2, IL-15	18.25 days; 20.5 days	–	No	Yes	–	2.5–10× 10^7^; 5–20× 10^7^	5 CR 2 PR 1 PD; 8 CR
Lee, D. W. (2015) [31]	21	20: ALL 1: NHL	Relapsed refractory	CD19	CD28, CD3ζ	2nd	Autologous	11 days	Retrovirus	CD3/CD28 beads	68 days	Fludarabine, cyclophosphamide	No	No	–	0.03–3× 10^6^/kg	14 CR 4 PD 3 SD
Kalos, M. (2011) [12]	3	CLL	Relapsed refractory	CD19	CD137 CD3ζ	2nd	Autologous	10 days	Lentivirus	CD3/CD28 beads	≥ 6 months	Bendamustine, rituximab, Pentostatin, cyclophosphamide	No	No	–	0.14–11× 10^8^	2 CR 1 PR

When counted the infusion cell number, the patients’ weight were identified as 50 Kg on average, and patients’ body surface area were identified as 1.8 on average

ALL: acute lymphocytic leukemia; CLL: chronic lymphocytic leukemia; FL: follicular lymphoma; MCL: mantle cell lymphoma; DLBCL: diffuse large B-cell lymphoma; NHL: non-Hodgkon’s lymphoma

LCLs: EBV-transformed lymphoblastoid B-cell lines

CR: complete response; PR: partial response; SD: stable disease; PD: progress disease; NR: no response; NE: not evaluate

### Efficacy

#### Response rate

A total of 178 patients were eligible for the response rate evaluation. The overall response rate was 67% (95%CI: 53–79%) (Table 2). Subgroup analyses were performed, and the results were showed in Table 2. We observed that patients who received lymphodepletion had higher response rate (72%; 95%: 63–80%; *P* = 0.0405) than patients who did not (44%; 28–62%) (Additional file 1: Figure S1). Patients whose peak serum IL-2 level was over50 pg/mL had higher response rate (85%; 95%: 55–96; P = 0.04) than those less than50 pg/mL (31%; 95%: 6–74%) (Additional file 1: Figure S2). Results of other subgroup analyses were presented in Table 2.

**Table 2:** Subgroup analyses of response rate

prognostic factor	events	n	I^2^	response rate(%)	95%CL	Q	*p*
Overall	125	178	0.584	67	53–79		
Ag recognition moieties							
CD19	118	169	62.6%	66	50–79		
CD20	7	9	0%	70	39–89	0.05	0.8187
Disease							
leukemia	90	125	50.3%	68	53–80		
lymphoma	35	53	53.8%	61	53–77	0.21	0.6482
T cell origin							
Autologous	116	157	53.9%	71	56–82		
Allogeneic	9	21	50.7%	46	17–78	1.74	0.1873
Generation							
1st	8	12	73%	61	7–97		
2nd	116	159	55.7%	69	56–80	0.07	0.7928
costimulatory domains							
CD137 and CD3ζ	49	63	36.1%	73	60–83		
CD28 and CD3ζ	68	101	59.9%	65	45–80	0.52	0.4715
T cell activation							
OKT3	86	105	42%	77	67–85		
CD3/CD28 beads	29	51	58%	56	31–79	2.91	0.0882
IL-2 administration to cells							
yes	42	75	67.5%	51	28–75		
no	78	97	17.9%	77	65–85	3.62	0.057
Transfection methods							
non-viral vector	2	5	4%	42	12–79		
viral vector	123	173	61%	69	54–80	1.41	0.2345
Lymphodepletion							
yes	98	127	34.1%	72	63–80		
no	15	38	42.1%	44	28–62	4.2	0.0405
CART cells							
≥ 10^8^	83	109	50.5%	72	56–84		
< 10^8^	36	50	6.5%	66	52–78	0.31	0.5782
IL-2 administration to patients							
yes	9	11	0%	72	44–90		
no	122	167	67.9%	67	49–81	0.12	0.7293
T cell persistence time							
≥ 2 months	92	117	0%	74	65–81		
< 2 months	34	60	56.4%	50	27–73	3.59	0.0581
Peak serum IL-2 level							
≥ 50 pg/mL	11	12	0%	85	55–96		
< 50 pg/mL	5	16	56.6%	31	6–74	4.22	0.04

#### Survival outcome

Progression free survival analysis included overall 90 patients from 15 clinical trials. The 6-month and 1-year PFS for this cohort were 65.62% (95%CL: 54.62–74.58%) and 44.18% (95%CL: 32.97–54.81%), respectively (Additional file 1: Figure S3A). The median and mean intervals of PFS were 10.4 and 21.62 (95%CL: 16.19–27.05) months, respectively. Association between patients’ PFS of CAR T cells immunotherapy and possible prognostic factors in univariate analysis were showed in Table 3. We observed that only CAR T cell costimulatory domains were related with PFS (*p* = 0.0489). The 1-year PFS of CD28 and CD3ζ (56.29%, 95%CL: 39.42–70.14%) was higher than that of CD137 and CD3ζ (33.39%, 95%CL: 16.56–51.22%) (Additional file 1: Figure S3B). The logrank test of other factors were showed in Table 3. Cox analysis showed that none factor was related to prognosis (Additional file 1: Table S1).

**Table 3:** Univariate analysis of patients’ PFS of CAR T cells immunotherapy and possible prognostic factors

prognostic factor	case(n)	Median PFS (months)	Mean PFS (months, 95%CL)	1-year PFS (%, 95%CL)	*p*-value
Ag recognition moieties					
CD19	81	10	24.11*(18.35–29.87)	46.12%(34.20–57.22%	
CD20	9	12	11.5(6.61–16.39)	33.33%(7.83–62.26%)	0.3309
Disease					
leukemia	42	7	20.30*(12.56–28.04)	40.19%(24.41–55.47%)	
lymphoma	48	12	18.10*(13.37–22.82)	48.22%(32.68–62.14%)	0.3123
T cell origin					
Autologous	74	12	22.33*(16.62–28.04)	45.60%(33.47–56.92%)	
Allogeneic	16	3	8.41*(5.23–11.59)	47.73%(22.05–69.64%)	0.1779
Generation					
1st	11	10.4	18.52*(9.86–27.18)	45.45%(16.66–70.69%)	
2nd	76	10	22.69*(16.40–28.98)	45.41%(33.11–56.91%)	0.7754
costimulatory domains					
CD137 and CD3ζ	28	6	16.44*(8.41–24.46)	33.39%(16.56–51.22%)	
CD28 and CD3ζ	46	–	14.50*(11.63–17.37)	56.29%(39.42–70.14%)	0.0489
T cell activation					
OKT3	43	12	12.76(9.80–15.73)	40.32%(23.45–56.63%)	
CD3/CD28 beads	34	12.6	25.02*(16.78–33.26)	52.78%(34.90–67.84%)	0.3961
IL-2 to cells					
yes	57	12.6	18.91*(14.15–23.67)	50.10%(35.63–62.95%)	
no	28	12	18.60*(10.86–26.34)	38.27%(19.56–56.81%)	0.616
transfection methods					
non-viral vector	6	12	12.83(7.72–1.94)	33.33%(4.61–67.56%)	
viral vector	94	10	23.99*(18.34–29.64)	45.75%(34.12–56.63%)	0.4634
Lymphodepletion					
yes	53	10	18.58*(12.16–24.99)	39.07%(25.16–52.72%)	
no	21	5	8.18*(5.49–10.87)	37.25%(12.81–62.22%)	0.3282
CART cells					
≥ 10^8^	54	8	21.43*(14.34–28.51)	42.01%(28.04–55.35%)	
< 10^8^	23	–	30.15*(20.10–40.20)	58.38%(34.69–76.06%)	0.1471
IL-2 administration to patients					
yes	13	12	13.44(9.19–17.70)	29.92%(7.49–57.01%)	
no	77	10	23.05*(16.19–27.05)	47.06%(34.99–58.22%)	0.9355
T cell persistence time					
≥ 2 months	44	10	18.33*(11.34–25.32)	37.26%(21.95–52.59%)	
< 2 months	46	12.6	18.82*(13.56–24.09)	50.62%(34.60–64.60%)	0.2986
Peak serum IL-2 level					
≥ 50 pg/mL	8	12	12*(7.84–16.16)	41.67%(7.20–74.73%)	
< 50 pg/mL	8	9	7.78*(3.61–11.94)	26.25%(1.27–66.37%)	0.4159

(*) largest observed analysis time is censored, mean is underestimated

### Safety

A total number of 154 patients were included in the overall analysis since two articles did not provide the data of the number of people with adverse events. The pooled estimate for overall incidence of any adverse events was 71% (95%CI: 0.49–0.92) (Additional file 1: Table S2). The estimate for incidence of grade ≥ 3 adverse events was 43% (95%CI: 0.23–0.63) within the related 154 patients (Additional file 1: Table S2).

**Fig. 3:**
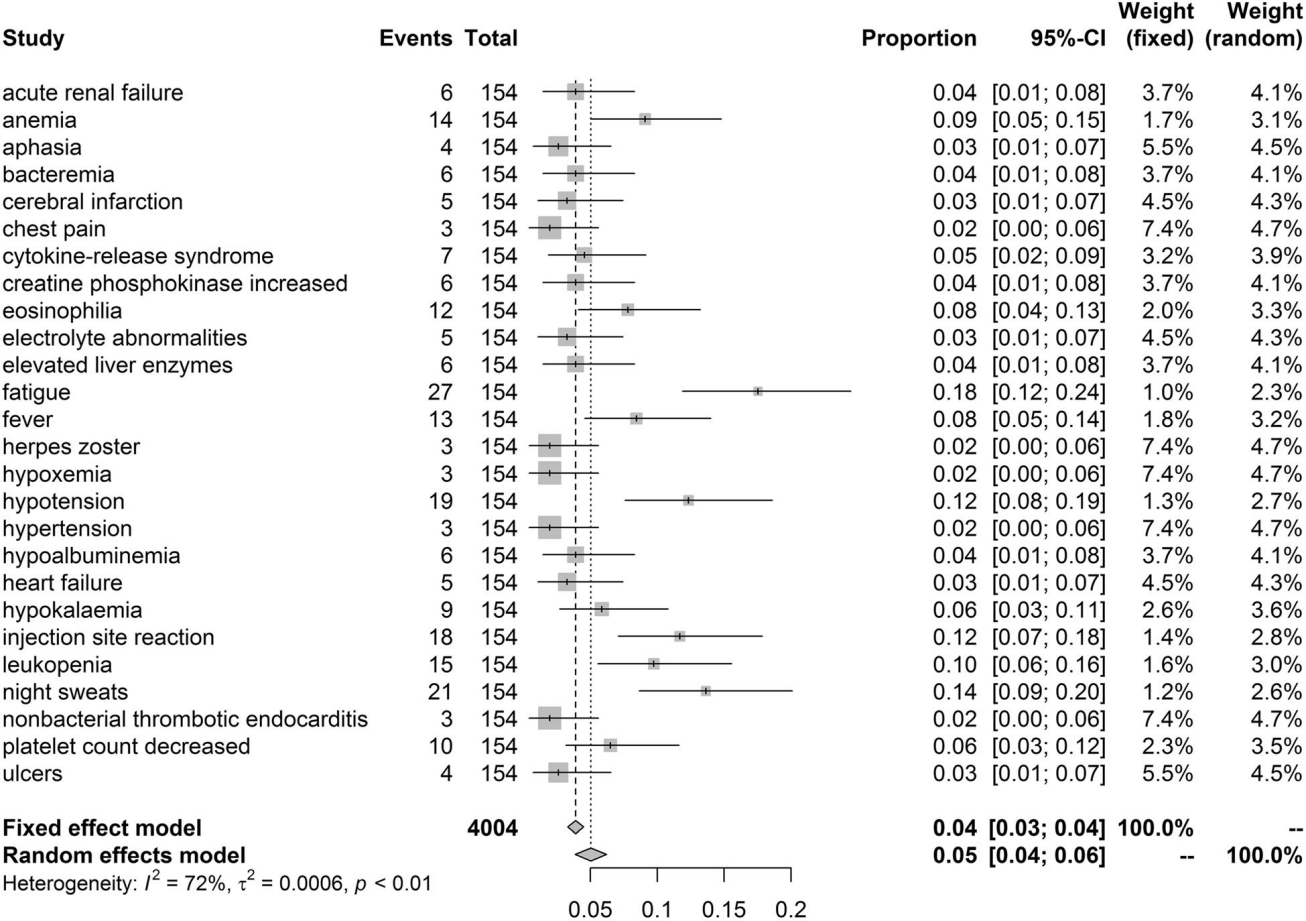
Forest plot for most common adverse events and confidence internals

After investigating grade ≥ 3 adverse events, we found that the most frequently occurred events included fatigue (18%, 95%CI: 0.12–0.24), night sweats (14%, 95%CI: 0.09–0.20), hypotension (12%, 95%CI: 0.08–0.19), injection site reaction (12%, 95%CI: 0.07–0.18), leukopenia (10%, 95%CI: 0.06–0.16), anemia (9%, 95%CI: 0.05–0.15) (Fig. 3).

By subgroup analysis, we did not discover that serum IL-2, IFN-γ and TNF levels were correlated to the incidence of toxicities (Additional file 1: Table S2).

### Publication bias

No potential publication bias was observed in funnel plot (Additional file 1: Figure S4).

## Discussion

CAR T cells immunotherapy is rapidly developed in recent decades. How to improve the efficacy and reduce treatment toxicity remains the most concerned issues. Therefore, the following processions need to be improved: CAR design, gene transfection method, cytokine support, expansion and persistence of T cells, patients’ preconditioning, infusion dose of T-cells and types.

According to signaling domains, there were first, second and third generations of CAR. Data suggested that second-generation CARs with a costimulatory molecule mediated rapid activation, expansion, and persistence to T cells compared with first generation CARs^,^ [19]. We discovered that the second-generation CAR T had a longer mean progression free survival time than first generation, but no significant difference(22.69 vs 18.52 months, *P* = 0.7754). Meanwhile, we didn’t find the difference in response (*P* = 0.7928) between first and second CARs. Therefore, the efficacy of second generation needs more research to verify. Because of the limited data, third generation was not evaluated. Whether third generation CARs are better than second generation CARs remains to be elucidated.

The costimulatory domain with second generation CAR T were usually used CD28 or CD137. Which domain shows better efficacy remains unknown. We discovered that no significant difference in the response rate between CD28 and CD137, but the CD137 signaling moieties in CARs related with lower survival (*p* = 0.0489). However, some studies exhibited that compared to CD28, the CD137 increased expansion and persistence of T cells [20, 21]. There were two possible reasons: first, CD137 was more novel, lacking of maturity; second, CD137-containing CARs could increase acute toxicity and the persistence of the infused T cells. There was no trial to compare the efficacy of costimulatory signal, therefore both basic and clinic trials are needed in this aspect.

CAR construction transducted to T cells by viral vector or electroporation. Viral transduction methods have higher transduction efficiency compared to electroporation, but it increases the risk of viral insertional oncogenesis. In our study, we did not find difference between the two methods. Considering only 5 patients transduced by electroporation, more trails are needed to detect gene transfer efficiency.

Should patients receive lymphodepletion or not, there was not been a common consensus by most researchers yet. Lymphodepletion regimen means depletion of recipient lymphocytes before CAR T cells infusion including chemotherapy, chemoradiotherapy, and monoclonal antibodies. It increased expansion, persistence, and efficacy of CAR T cells by eliminating regulatory T cells and other immune cells that may compete for cytokines, including IL-15 and IL-7, which activating antigen-presenting cell [22–24]. In this study, lymphodepletion was associated with better response (*P* = 0.0405), but no evidence of correlation with PFS, the same with the former article [15]. However, we didn’t perform subgroup analysis to assess the efficacy between different lymphodepletion regimens. In the future, research should focus on the effect of different lymphodepletion regimen on patients received CAR T cells.

Cytokine were often added to expanse T cells. Previous study presented that IL-2 promoted T-cell expansion to affect the efficacy [25]. We observed that peak serum IL-2 level in patients (P = 0.04) were positively associated with patients’ response to CAR T cells, in accordance with previous study. However, we observed that whether IL-2 administration to T cells or patients or not, the efficacy had no difference, not in accordance with former study [15]. These were two possible reasons for this result: first, the costimulatory domain could active antigen specific cytokine production cells without IL-2. Second, anti-CD3/anti-CD28 mAb-coated magnetic beads can stimulate T cell expansion without IL-2. Therefore, whether IL-2 administration to T cells or patients or not still needs more studies.

After infusion of CAR T cells, the cells will expanse to play a role and then go to apoptosis. Degree of expansion and duration of persistence is often considered to correlate with efficacy [26, 27]. However, we didn’t observe that expansion and persistence of T cells were related with efficacy. The following reasons should be considered for the result. First, previous study observed that costimulatory domain can increase persistence [28]. Next, other studies showed that lymphodepletion was benefitial to T-cell persistence and expansion in vivo [29, 30]. Meanwhile, IL-2 promoted T-cell expansion [25]. All these factors can influence efficacy. Consequently, during the process of CAR T therapy, more attention are needed to be paid in these procedures.

Commonly, the efficacy correlated with drug dose. There was no standard infusion dose of CAR T cells. Previous study defined the maximum tolerated CAR T cells dose as 1X 10^6^ CAR T cells/ kg body weight [31]. The only existing reports failed to identify a correlation of transfused CAR T cells number and clinical efficacy. Also, the dose of administered CAR T cells could not predict peak blood levels of CAR T cells [12, 14] . These results were in accordance with our finding. We assume the reasons behind this may be that there were regulatory T cells repressed expansion in vivo. Meanwhile, interindividual variation may make significant differences.

Mature Th cells express the surface protein CD4 and are referred to as CD4+ T cells. They function in the activation of other immune cells by releasing T cell cytokines. Cytotoxic T cells killed virus-infected cells and tumor cells, and they are also related to transplant rejection. These cells are known as CD8+ T cells since they express the CD8 glycoprotein. Several studies observed that CD4+ and CD8+ contents and the proportion of T cells may affect efficacy [4, 32]. However, previous study reported that the absolute numbers of infused T-cell subsets did not appear to relate with clinical efficacy [4]. Our study didn’t analyze the proportion of CD4+/CD8+ whether related with efficacy with limited data. Further researches need be explored to find the optimal strategies.

Toxicity included CRS, on-target off-tumor effects and the toxicity caused by lymphodepletion. CRS can be caused by massive therapy-induced release of inflammatory cytokines. On-target off-tumor effects destroyed normal cells with the CAR-targeted antigens. We observed that the overall incidence of any adverse events was 71%, incidence of grade ≥ 3 adverse events was 43%, the most frequently occurred events included fatigue (18%), night sweats (14%), hypotension (12%), injection site reaction (12%) among the grade ≥ 3 adverse events. In patients after CAR T-cell infusion, IFN-γ and TNF are commonly high, which induces sepsis-like syndrome and causes organ failure [13]. However, these were not in accordance with our results. But we found that adverse events with higher IL-2, TNF, IFN-γ cytokine level happened more frequently. These factors were also closely related to CAR T-cell antitumor activity. Therefore, how to balance the efficacy and the toxicity should be further considered. A suicide gene, inducible caspase 9 (iCasp9) was integrated to CAR construction to regulate the persistence of CAR T-cells to control the on-target/off-tumor toxicities [33].

We included 18 articles to assess the efficacy and safety of CD19 or CD20-CAR T cells immunotherapy. Furthermore, we detected the factors affecting the efficacy and safety of therapy. However, our study has several limitations. First, the included articles were not totally prospective clinic studies, the potential performance bias might exist. Second, more studies were needed to assess the efficacy and sefety of CAR T therapy.

## Conclusions

In conclusion, our study demonstrated a high response rate of CAR T therapy in refractory B cell malignancies. The study also showed lymphodepletion regimen and high serum IL-2 level were associated with better clinical responses, and that costimulatory domains was related with better PFS. Further modifications of CAR structure and optimal therapy strategy in continuing clinical trials are needed to obtain significant improvements.

## Additional file

Additional file 1: **Figure S1.** Forest plot for response rates and confidence internals in patients with or without lymphodepletion. **Figure S2.** Forest plot for response rates and confidence internals in patients with different serum IL-2 level. **Figure S3.** Progression-free survival (PFS) curves. A. the PFS for 90 patients; B. patients received CAR T cells with CD28 costimulatory domain had better PFS than CD137. **Figure S4**. funnel plot of substantial publication bias. **Table S1.** Cox regression analysis of patients’ PFS of CAR T cells immunotherapy and possible prognostic factors. **Table S2.** Subgroup analyses of adverse events. (DOCX 924 kb)

## Abbreviations

AEs: Adverse effects
ALL: Acute lymphocytic leukemia
CAR T: Chimeric antigen receptor- T cells
CLL: Chronic lymphocytic leukemia
CM: Costimulatory molecule
CR: Complete response
CRS: Cytokine release syndrome
DLBCL: Diffuse large B-cell lymphoma
FL: Follicular lymphoma
ITAMs: Immunoreceptor tyrosine-based activation motifs
LCLs: EBV-transformed lymphoblastoid B-cell lines
MCL: Mantle cell lymphoma
MHC: Major histocompatibility compleX
NHL: Non-Hodgkon’s lymphoma
NR: No response; NE: not evaluate
PD: Progress disease
PR: Partial response
scFv: Single-chain variable fragment
SD: Stable disease
TAA: Tumor associated antigen
TCR: T cell receptors
