# Supplementary material for: The efficacy and safety of anti-CD19/CD20 chimeric antigen receptor- T cells immunotherapy in relapsed or refractory B-cell malignancies:a meta-analysis
Source: BMC Cancer. 2018 Sep 26;18:929. doi: 10.1186/s12885-018-4817-4 (PMC6158876; doi:10.1186/s12885-018-4817-4)
Supplement: Supplementary file 1 — Figure S1. Forest plot for response rates and confidence internals in patients with or without lymphodepletion. Figure S2. Forest plot for response rates and confidence internals in patients with different serum IL-2 level. Figure S3. Progression-free survival (PFS) curves. A. the PFS for 90 patients; B. patients received CAR T cells with CD28 costimulatory domain had better PFS than CD137. Figure S4. funnel plot of substantial publication bias. Table S1. Cox regression analysis of patients’ PFS of CAR T cells immunotherapy and possible prognostic factors. Table S2. Subgroup analyses of adverse events. (DOCX 924 kb) [file 12885_2018_4817_MOESM1_ESM.docx]

**The efficacy and safety of anti-CD19/CD20** **chimeric antigen receptor- T cells immunotherapy in** **relapsed or refractory B-cell malignancies**

Hui Zhou^1^, Yuling Luo^1^, Sha Zhu^1^, Xi Wang^1^, Yunuo Zhao^1^, Xuejin Ou^1^, Tao Zhang^1^, Xuelei Ma^1,^*.


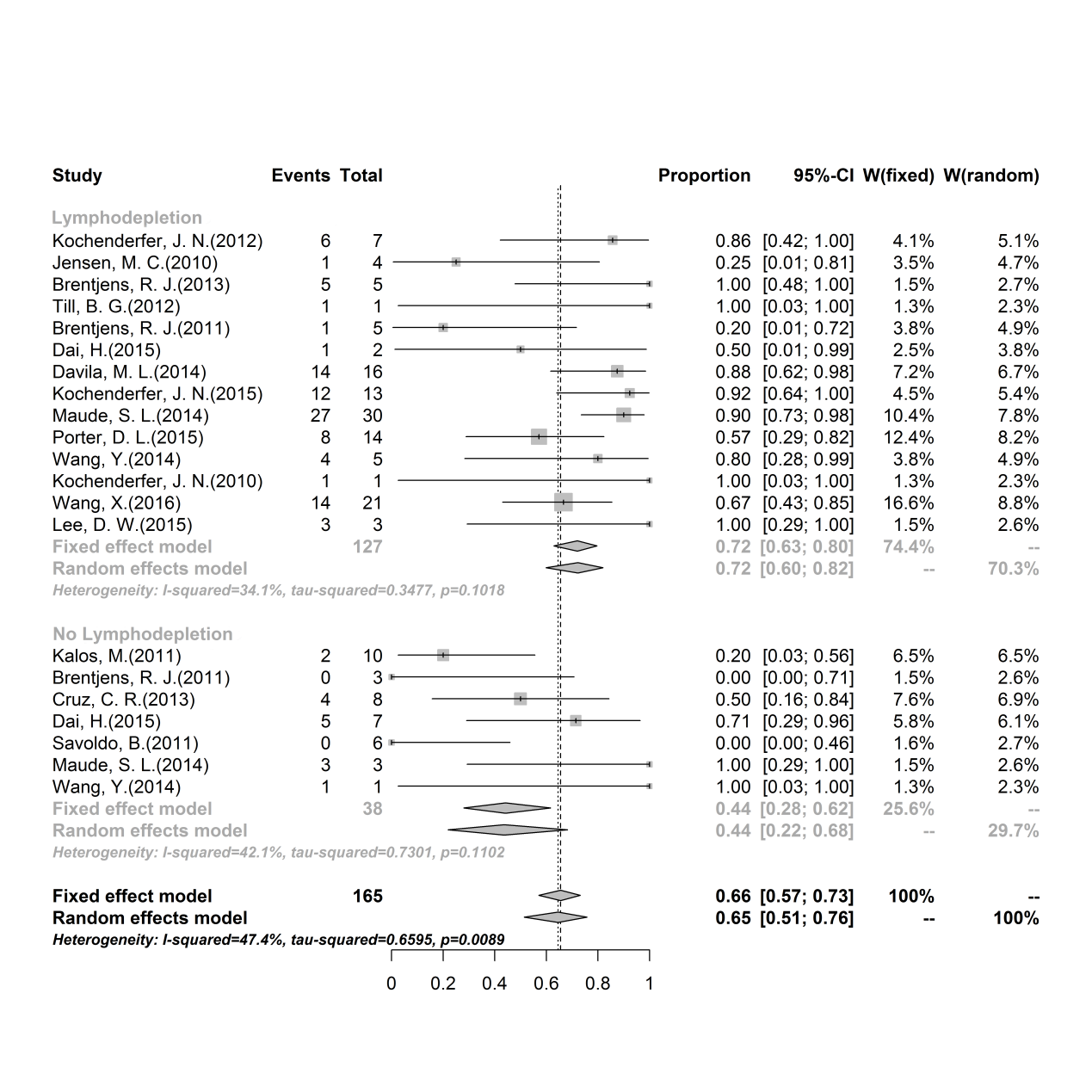


**Additional file 1: Figure S1: Forest plot for response rates and confidence internals in patients with or without lymphodepletion.**

**
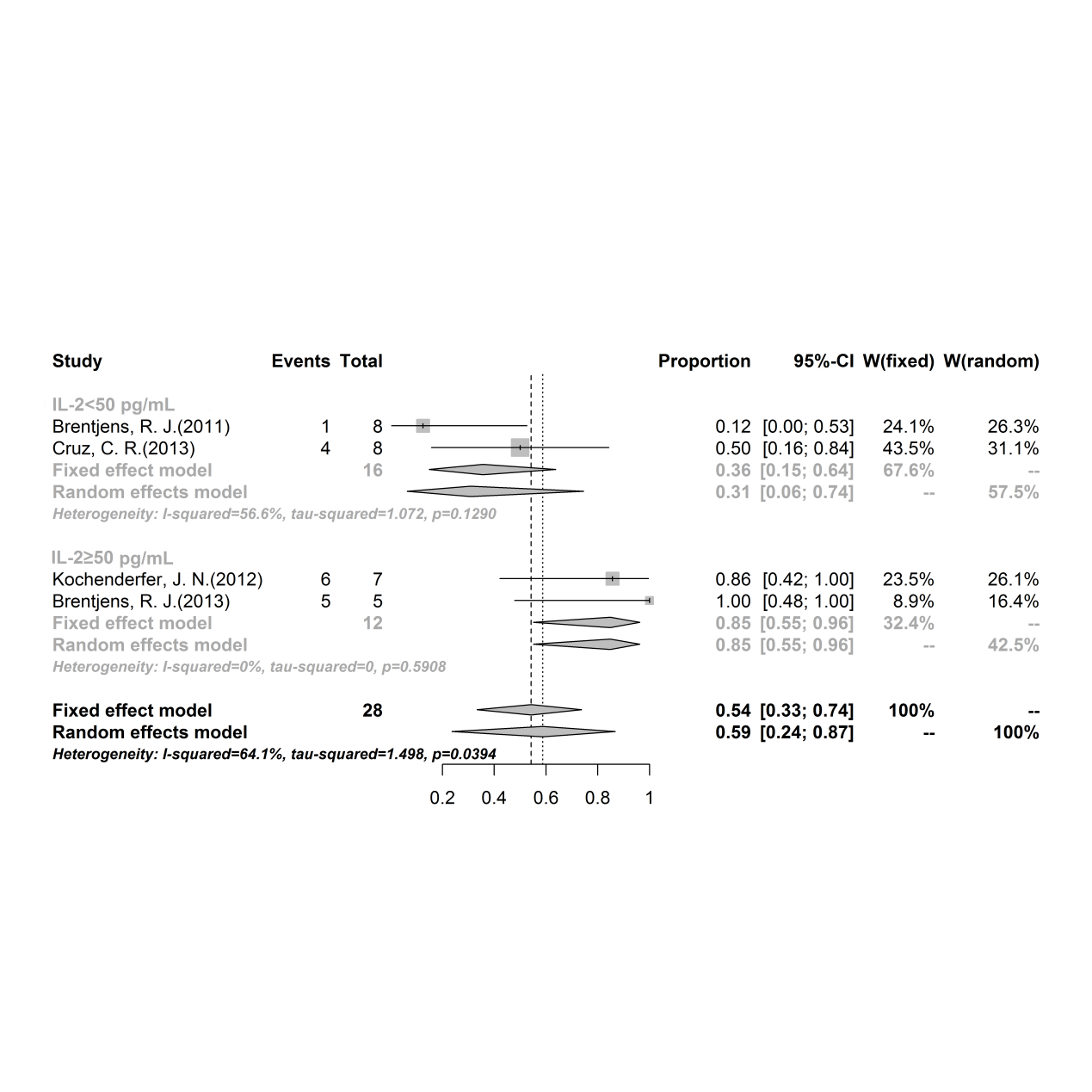
**

**Additional file 1: Figure S2: Forest plot for response rates and confidence internals in patients with different serum IL-2 level.**

**
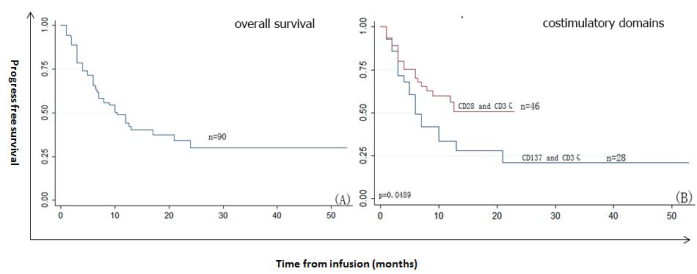
**

**Additional file 1: Figure S3: Progression-free survival (PFS) curves. A. the PFS for 90 patients; B. patients received CAR T cells with CD28 costimulatory domain had better PFS than CD137.**


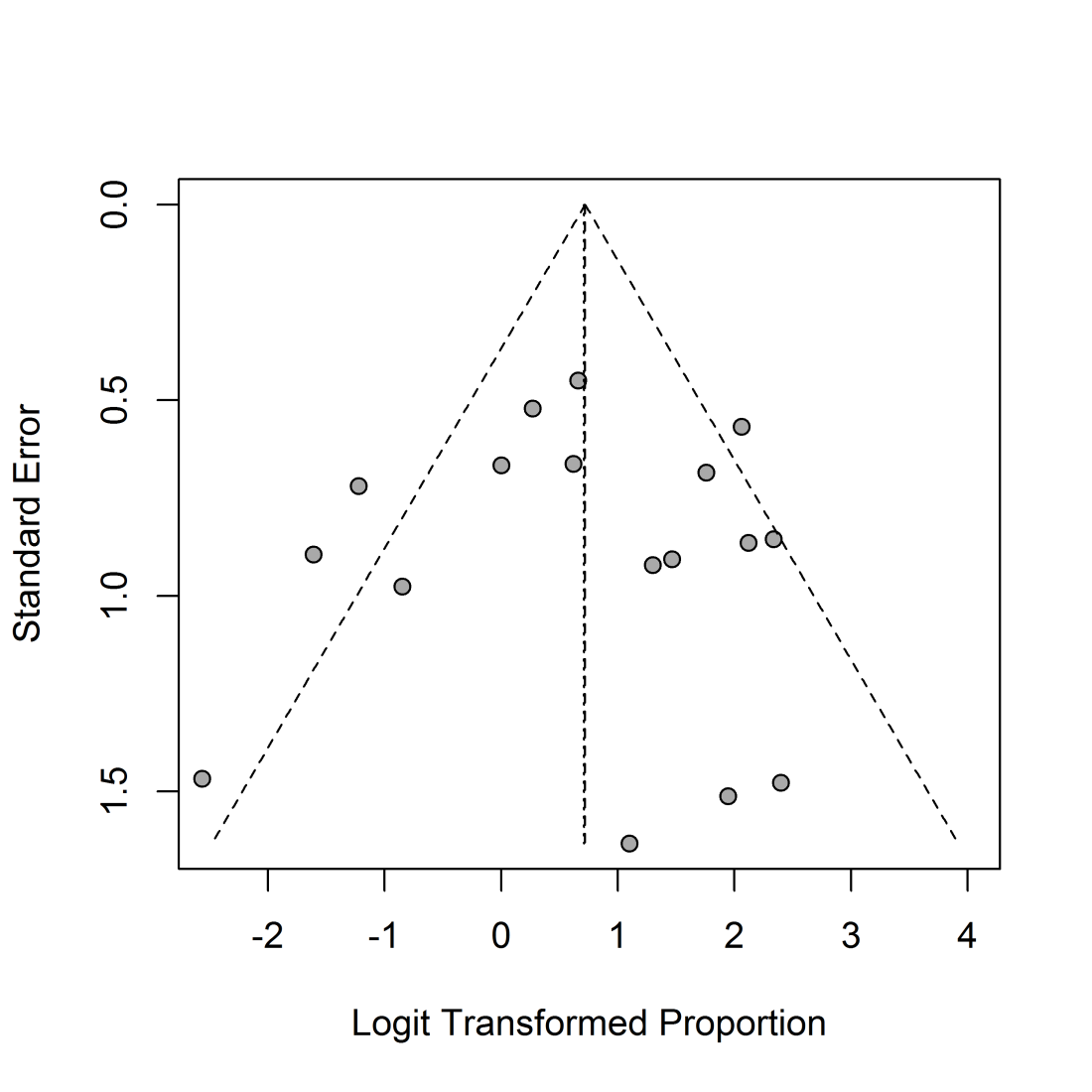


**Additional file 1: Figure S4.** **funnel plot of substantial publication bias.**

| **Additional file 1: Table S1. Cox regression analysis of patients’ PFS of CAR T cells immunotherapy and possible prognostic factors** | | | | | |
| --- | --- | --- | --- | --- | --- |
| **prognostic factor** | **case(n)** | **Haz. Ratio (95%cl)** | **Std. Err.** | **z** | **P>\|z\|** |
| **Ag recognition moieties** | 90 | 1.44(0.68-3.07) | 0.56 | 0.94 | 0.345 |
| **Disease** | 90 | 0.76(0.44-1.32) | 0.21 | -0.98 | 0.325 |
| **T cell origin** | 90 | 1.67(0.77-3.61) | 0.66 | 1.3 | 0.194 |
| **Generation** | 87 | 0.46(0.50-2.52) | 0.46 | 0.28 | 0.78 |
| **costimulatory domains** | 74 | 0.55(0.29-1.02) | 0.18 | -0.88 | 0.059 |
| **T cell activation** | 77 | 0.77(0.42-1.42) | 0.24 | -0.83 | 0.406 |
| **IL-2 to cells** | 85 | 1.16(0.65-2.08) | 0.34 | 0.49 | 0.623 |
| **Transfection methods** | 90 | 0.73(0.31-1.72) | 0.32 | -0.71 | 0.475 |
| **Lymphodepletion** | 74 | 1.39(0.70-2.80) | 0.5 | 0.94 | 0.349 |
| **CART cells** | 77 | 0.60(0.29-1.23) | 0.22 | -0.14 | 0.16 |
| **IL-2 administration to patients** | 90 | 0.97(0.47-2.00) | 0.36 | -0.08 | 0.937 |
| **T cell persistence time** | 90 | 0.75(0.43-1.30) | 0.216 | -1.01 | 0.312 |
| **Peak serum IL-2 level** | 16 | 1.765(0.435-7.169) | 1.262 | 0.79 | 0.427 |

| **Additional file 1: Table S2. Subgroup analyses of adverse events.** | | | | | | | |
| --- | --- | --- | --- | --- | --- | --- | --- |
| **prognostic factor** | **events** | **n** | **I^2^** | **adverse events(%)** | **95%CL** | **Q** | ***p*** |
| **Overall** | 120 | 159 | 98% | 71 | 49-92 |  |  |
| **grade ≥ 3** | 64 | 154 | 96% | 43 | 23-63 |  |  |
| **Peak serum IL-2 level** | | |  |  |  |  |  |
| ≥50 pg/mL | 13 | 13 | 0% | 93 | 64-99 |  |  |
| <50 pg/mL | 9 | 17 | 87% | 51 | 0-1 | 0.7 | 0.4038 |
| **Peak serum TNF level** | | |  |  |  |  |  |
| ≥50 pg/mL | 17 | 17 | 0% | 95 | 71-99 |  |  |
| <50 pg/mL | 13 | 23 | 87% | 40 | 1-98 | 1.76 | 0.1851 |
| **Peak serum IFN-γ level** | | |  |  |  |  |  |
| ≥200 pg/mL | 19 | 26 | 66% | 66 | 17-95 |  |  |
| <200 pg/mL | 3 | 23 | 73% | 20 | 1-56 | 1.06 | 0.3024 |
